# The daily updated Dutch national database on COVID-19 epidemiology, vaccination and sewage surveillance

**DOI:** 10.1038/s41597-023-02232-w

**Published:** 2023-07-20

**Authors:** E. L. P. E. Geubbels, J. A. Backer, F. Bakhshi-Raiez, R. F. H. J. van der Beek, B. H. B. van Benthem, J. van den Boogaard, E. H. Broekman, D. A. Dongelmans, D. Eggink, R. D. van Gaalen, A. van Gageldonk, S. Hahné, K. Hajji, A. Hofhuis, A. J. van Hoek, M. N. Kooijman, A. Kroneman, W. Lodder, M. van Rooijen, W. Roorda, N. Smorenburg, F. Zwagemaker, National sewage surveillance group, Yu-Ling Beck, Dorothe van Beugen, Michiel van Boven, Titus Breuning, Chesley van Buuren, Sipke Dijkstra, Weiyi Ding, Anne-Merel van der Drift, Ivo Grift, Auke Haver, Wouter Hetebrij, Demi van de Hoef, Kim de Jong, Arnoud de Klijne, Jaap Koelewijn, Jannetje Kooij, Jeroen Korevaar, Gretta Lynch, Erwin Nagelkerke, Süeda Nicanci, Noel Peters, Céline Peterse, Rozemarijn van der Plaats, Elsa Poorter, Gino Raaijmakers, Lars van Rijckevorsel, Sharona de Rijk, Nathanaël Sam-Sin, Merve Senyer, Reza Sheikh Moghaddas, Sjors Stouten, Rick Theijn, Max van Velzen, Ilse Voshart, Anne Welling, Arno Wijsmuller, Nicolas Winkelhorst, Gimairo Wong-Loi-Sing, RIVM COVID-19 epidemiology, surveillance team, Stijn Andeweg, Patrick van den Berg, Danytza Berry, Bronke Boudewijns, Siméon de Bruijn, Kirsten Bulsink, Thomas Dalhuisen, Senna van Iersel, Liz Jenniskens, Femke Jongenotter, Marit de Lange, Susan Lanooij, Hester de Melker, Amber Maxwell, Nienke Neppelenbroek, Steven Nijman, Priscila de Oliviera Bressane Lima, Fleur Petit, Tara Smit, Anne Teirlinck, Anne-Wil Valk, Irene Veldhuijzen, Carolien Verstraten, Lieke Wielders, Guido Willekens, N. F. de Keizer, I. van Walle, A. M. de Roda Husman, C. Ruijs, S. van den Hof

**Affiliations:** 1 Centre for Infectious Diseases Control, National Institute for Public Health and the Environment, Bilthoven, the Netherlands; 2 National Intensive Care Evaluation (NICE) foundation, Amsterdam, the Netherlands; 3 The network of regional epidemiological consultants (REC), Bilthoven, the Netherlands; 4 GGD GHOR Nederland, Utrecht, the Netherlands; 5 Amsterdam UMC, University of Amsterdam, Amsterdam, the Netherlands; 6 Centre of Information Services and CIO office, National Institute for Public Health and the Environment, Bilthoven, the Netherlands; 7 Department of Medical Informatics, Amsterdam UMC, Amsterdam Public Health research institute, University of Amsterdam, Amsterdam, the Netherlands

**Keywords:** Viral infection, Viral genetics, Epidemiology, Epidemiology, Risk factors

## Abstract

The Dutch national open database on COVID-19 has been incrementally expanded since its start on 30 April 2020 and now includes datasets on symptoms, tests performed, individual-level positive cases and deaths, cases and deaths among vulnerable populations, settings of transmission, hospital and ICU admissions, SARS-CoV-2 variants, viral loads in sewage, vaccinations and the effective reproduction number. This data is collected by municipal health services, laboratories, hospitals, sewage treatment plants, vaccination providers and citizens and is cleaned, analysed and published, mostly daily, by the National Institute for Public Health and the Environment (RIVM) in the Netherlands, using automated scripts. Because these datasets cover the key aspects of the pandemic and are available at detailed geographical level, they are essential to gain a thorough understanding of the past and current COVID-19 epidemiology in the Netherlands. Future purposes of these datasets include country-level comparative analysis on the effect of non-pharmaceutical interventions against COVID-19 in different contexts, such as different cultural values or levels of socio-economic disparity, and studies on COVID-19 and weather factors.

## Background & Summary

The COVID-19 pandemic has so far claimed at least 6 million deaths and, at the time of writing, mid-August 2022, is still causing over 3.5 million cases and almost 10,000 deaths weekly (https://covid19.who.int/). In the Netherlands, more than 8 million cases have been reported since the first case was identified on 27 February 2020 (https://www.rivm.nl/en/node/163991) and close to 40,000 people died from COVID-19 in 2020 and 2021 (https://www.cbs.nl/nl-nl/longread/rapportages/2022/sterfte-en-oversterfte-in-2020-en-2021).

As part of its mandate to promote and protect public health, the National Institute of Public Health and the Environment (RIVM) in the Netherlands organizes the national surveillance of notifiable diseases. The RIVM adheres to principles of Open Science, including open access to (meta)data. In providing open data, the adagio “open where possible, restricted where required” is used to strike a balance between granting maximum access to data for re-use while protecting the privacy rights of Dutch citizens^1^.

Prior to the COVID-19 pandemic, a well-oiled surveillance system for notifiable diseases already existed in the Netherlands, which is a close collaboration between the national public health institute, municipal health services (MHSes), laboratories and hospitals. In response to COVID-19, surveillance was intensified to include novel data sources and more real-time information-gathering. Data analysis and reporting were automated to facilitate daily data updates and information-sharing through open datasets and detailed dashboarding (https://coronadashboard.rijksoverheid.nl/).

Throughout the pandemic, these COVID-19 surveillance data have been used in the Netherlands and in international collaborations for estimating viral transmission characteristics^2–4^, targeting local outbreak control measures (https://www.rstudio.com/blog/how-the-clusterbuster-shiny-app-helps-battle-covid-19-in-the-netherlands/), identification of at-risk groups^5–8^, planning for health system capacity, public information in burden of disease (https://www.rivm.nl/en/coronavirus-covid-19/weekly-figures), sewage surveillance (https://www.rivm.nl/en/covid-19/sewage) and virus variants (https://www.rivm.nl/en/coronavirus-covid-19/virus/variants) and the planning for and evaluation of vaccination programs and non-pharmaceutical interventions against COVID-19, such as work from home recommendations and closure of educational facilities, pubs and restaurants^9–12^.

At the time of writing, a suite of open datasets related to the epidemiology of COVID-19 is published, most of them every workday and at a sub-national level.

The aim of this data descriptor is to describe the underlying methods through which these real-time open data are generated, discuss data validity, describe (meta)datasets and where to find them, present key results and explore options for re-use by others.

## Methods

**Fig. 1:**
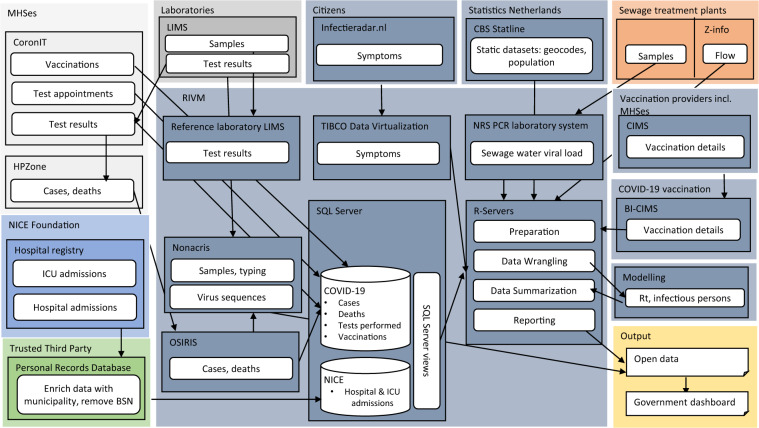
Overview of the data sources, main content and data flow for the open data sets in the national Dutch COVID-19 epidemiological surveillance. Names in larger lighter shaded box refer to the entities generating data, names in smaller darker shaded box refer to the data system. Box colors indicate the custodians of the source data: light grey=MHS, blue=NICE foundation, green=Trusted Third Party, medium grey=laboratories, dark grey= RIVM, orange= Sewage Treatment Plants. The yellow box contains the output dataset.

The current national epidemiological surveillance system for COVID-19 from which open data are produced consists of 8 data sources, which are shown in Fig. 1. The system is described below in brief and in more detail under the separate headings in this Methods section.

In terms of disease information, MHSes report test appointments made and their laboratory results, as well as notifiable cases of COVID-19 with patient background information, hospitals report COVID-19 positive admissions to both general wards and ICUs, while citizens log the presence of COVID-19 related symptoms. Laboratories generate virus sequence information from PCR positive samples, which are deposited in a dataset on virus variant distribution. Sewage water samples are analyzed to quantify viral load. The number of infectious persons and the effective reproduction number are calculated from case notification and hospital admission data. Vaccination data are submitted by all vaccination providers, with MHSes constituting the largest providers.

Case notifications, hospital admissions, test appointments and results, and viral load in sewage water data have full coverage, whereas the data on virus variants is derived from a random sampling frame. Vaccination data have >90% coverage. Population-based COVID-19 symptoms data come from a non-random, self-selected sample of citizens.

Datasets obtained from these eight data sources are transmitted using various data platforms, all but one of which (i.e. CoronIT) existed prior to the COVID-19 pandemic and serve multiple infectious disease reporting requirements. Where necessary for calculation of disease estimates or for enrichment of data, these dynamic datasets are combined with static datasets on population numbers, geocodes and with dynamic sewage flow data. Through automated scripts (refer to section Technical Validation) all datasets are imported, validated, analyzed and reported, including as 18 open data sets, which are presented in Table S1, with their version number at the time of writing this Data Descriptor and described in more detail below. This epidemiological surveillance system published its first open dataset (the cumulative and cases by municipality) on 30 April 2020, and was progressively extended as more data sources became available.

The surveillance of SARS-CoV-2 and COVID-19 is governed by the RIVM Act (https://wetten.overheid.nl/jci1.3:c:BWBR0008289&z=2020-03-19&g=2020-03-19) and the Dutch privacy legislation, which fully adopts the European General Data Protection Regulation (GDPR)^1^.

Care has been taken that all data shared in open datasets constitutes anonymous data, i.e. it no longer qualifies as personal data according to GDPR. This is done by restricting or aggregating data such that individuals cannot reasonably be identified within the open data, nor from the combination of these datasets with other datasets and publicly available reports. For example, counts may be presented at a less granular geographical level than what is available in the source data. Where relevant, the steps through which this is done are indicated under each of the 8 data source headings below. Lastly, research that uses already collected data is not subject to the Medical Research Involving Human Subjects Act (WMO), and does not need to undergo medical ethical review (https://english.ccmo.nl/investigators/legal-framework-for-medical-scientific-research/your-research-is-it-subject-to-the-wmo-or-not).

### Case notifications

OSIRIS (Online System for Infectious disease Reporting within ISIS) is the backbone of infection control information exchange between MHSes and the RIVM for all notifiable diseases in the Netherlands, and has been used from the start of the SARS-CoV-2 pandemic.

#### Data collection

Upon notification of a case of SARS-CoV-2 by a medical doctor or laboratory, the MHS initiates source and contact-tracing interviews and registers the information in an Electronic Patient File system (HPZone (Lite), inFact Software UK Ltd). A selection of data from HPZone is transferred to the OSIRIS database at the RIVM, according to an evolving set of specifications. Currently (22 August 2022) the OSIRIS specifications are in their 10^th^ version, with contents having been adapted regularly to include information pertinent to the stage of the epidemic. E.g. over time, information about type of test (PCR of antigen test), details on institutional living setting, and vaccination history have been added. Case identification is through a unique identifier assigned to each reported case; the key linking the national personal identifier (“burgerservicenummer” or “BSN” in Dutch) and the person’s contact information remains with the MHSes.

As part of source and contact-tracing, the index is interviewed about underlying illnesses, onset and progression of COVID-19 symptoms, vaccination history, their living, working and educational situation, their whereabouts during the incubation period, and whether they have been in contact with someone with a confirmed COVID-19 infection. The latter two are asked in order to assess the possible source and setting of infection. Furthermore, persons exposed to the index in their infectious period, are identified as contacts. As far as this information is known to the MHS, hospital admissions and deaths are registered in the case notification data record, but because these are not notifiable by law, these data are known to be incomplete in OSIRIS. Therefore alternative methods are used for calculating the hospital admission rate (refer to section Hospital and ICU admissions) and the mortality impact of SARS-CoV-2, i.e. excess mortality estimation and cause-specific mortality analysis (https://www.cbs.nl/nl-nl/longread/rapportages/2022/sterfte-en-oversterfte-in-2020-en-2021).

The goals of contact-tracing are to identify and inform contacts of the infected case of their exposure and risk of infection, to inform them about infection prevention measures to reduce further spread, including testing and quarantine, and to support them in adhering to these measures. Furthermore, the surveillance data from contact tracing have been used to investigate routes and settings of transmission and to evaluate the effect of vaccination and other infection control interventions on transmission.

Until 21 January 2022, the intensity and reach of contact interviews depended on the case load and the context of the infection. The intensity levels of source and contact tracing were categorized as ‘high’: full source and contact tracing leading to complete information about possible settings of transmission; ‘medium’: intense contact tracing was only done for high-risk groups/areas, so information about settings is mainly derived from those groups; ‘low’: very limited contact tracing was done, information about settings is very incomplete. As of 21 January 2022, this system of standard source and contact tracing was abolished and MHSes now decide on a case-to-case basis whether to instigate source and contact tracing. For surveillance purposes, MHSes do still report a random sample of cases for which a full index interview is done. These are reported via the OSIRIS system, to allow interpretation of the described additional epidemiological characteristics of cases, such as vaccination status, and the setting of infection. The sampling fraction is proportional to the case load in an MHS, to a combined daily maximum of 750 sampled cases nationally. Throughout these changes in modality of contact tracing, index cases who are not phoned by the MHS have received written instructions and tools to help them inform their contacts.

To accommodate the very high number of cases caused by the Omicron variant, an additional data feed was created for MHSes and other SARS-CoV-2 test providers to report cases with a positive test directly to the RIVM from 8 February 2022 onwards, instead of the data feed through HPZone. Through this feed, only limited data are automatically reported about each case, and no follow-up of contacts is done by MHSes after case registration.

#### Variable definition

At the beginning of the epidemic in the Netherlands (27 February 2020), the initial case definition distinguished between suspected and confirmed cases. A suspected case was a person with a fever of at least 38 degrees Celsius, plus at least one of the following respiratory symptoms: coughing, shortness of breath AND whose symptoms started within 14 days after return from mainland China (excluding Hong Kong, Macau and Taiwan) OR within 14 days after contact with a patient with confirmed SARS-CoV-2 infection.

A confirmed case was a person with a positive SARS-CoV-2 RT-PCR test aimed at two independent viral targets, irrespective of their clinical symptoms or epidemiological criteria.

The suspected case definition was repeatedly adjusted to reflect the countries with established local transmission, until both this geographical epidemiological criterium and the epidemiological link with a confirmed case was removed from the suspected case definition on 11 March 2020. As of 20 March 2020, suspected cases were no longer notifiable.

The confirmed case definition was adapted two times: on 15 October 2020, with the addition of a positive laboratory-performed rapid antigen test validated in the Netherlands; and on 10 May 2021, with the addition of a positive self-administered rapid antigen tests.

#### Use in open data sets

Seven of the 18 open data sets on national epidemiological SARS-CoV-2 surveillance contain information solely from the OSIRIS system. These concern (1) the case-based register (Table S1, dataset 1)^13^; (2) the cumulative number of notified and deceased cases per municipality per day (Table S1, dataset 2)^14^; (3) the number of new cases and deaths per municipality per day (Table S1, dataset 3)^15^; (4) the daily number of new cases and deaths in disability care institutions, as well as number of locations with new and ongoing outbreaks, per security region (Table S1, dataset 4)^16^; (5) the daily number of new cases and deaths in nursing homes, as well as number of locations with new and ongoing outbreaks, per security region (Table S1, dataset 5)^17^; (6) the daily number of new cases and deaths among elderly (70 years and older) living at home, per security region (Table S1, dataset 6)^18^; and (7) the possible settings of transmission of newly notified cases, per day per security region – reported only when the intensity level of contact tracing is ‘medium’ (as per 8 November 2011) or ‘high’ (Table S1, dataset 7)^19^. To anonymize personal data in the case-based register open dataset, date of death is presented as week of death, and age is presented in 10 year age-groups (for living patients and deceased patients over 50 years), except for deceased patients under 50 years old, who are allocated to the age group of <50 years. To anonymize datasets 4, 5, 6 and 7, data are presented at security region level rather than municipal level.

### Hospital and ICU admissions

The National Intensive Care Evaluation (NICE) Foundation has facilitated the registration of COVID-19 patients admitted to hospital, including to the ICU, since mid-March 2020. Prior to and during the SARS-CoV-2 pandemic, NICE routinely registers, analyses and publishes information on the quality of care and patient outcomes from all 81 Dutch ICU’s^20^. In response to the COVID-19 pandemic, and because OSIRIS case notification data are incomplete with regards to hospitalizations, this registry was extended to include registration of patients admitted with COVID-19 in all Dutch hospitals with inpatient COVID-19 wards (n = 82) (https://www.stichting-nice.nl/COVID_rapport_afdeling.pdf).

#### Data collection

Hospitals report individual-level patient data to NICE either through an automated data extraction from their electronic patient file system or through a manual upload onto a NICE registry webserver. Data submitted by hospitals to NICE include the patient’s BSN identifier, admission and discharge dates of each episode in the general ward or at the ICU, the admission type (general ward or ICU), the vital status at discharge and -for ICU admissions only- whether the patient was transferred in from or out to another ICU and whether they were discharged to home, the hospital ward or a different hospital. NICE adds a registry-specific patient-ID. A Trusted Third Party provider enriches this data set with the patient’s municipality of residence from the national Personal Records Database, using the patient’s BSN identifier. These enriched data, excluding the BSN identifier, but including the NICE generated patient ID, are then sent to the RIVM. These data thus cannot be linked with other datasets, do not allow identification of specific individuals, but do enable the RIVM to determine whether a patient has been admitted previously.

#### Variable definition

A new hospital admission is counted when a SARS-CoV-2-infected patient is admitted to hospital for the first time, or when the patient’s admission date is at least 90 days after a previous admission date. Patients who are repeatedly transferred or re-admitted within one disease episode thus count as one admission. New hospital admissions include both admissions to the non-ICU ward and straight to the ICU. Similarly, but as a separate count, new ICU admissions are defined as the first ICU admission episode for each SARS-CoV-2-infected patient, or a repeat admission that happened at least 90 days after the previous admission date. SARS-CoV-2-infected patients are those with a PCR-confirmed infection or a classification of CO-RADS 4 or 5^21^, where the PCR test is decisive in case of conflicting diagnostic results. In practice the CO-RADS based definition was only used during the first wave in 2020 when there was a shortage of PCR testing.

New hospital admissions include patients who had an admission-indication other than COVID-19 but who happened to test positive for SARS-CoV-2, because their prognosis is negatively influenced by their SARS-CoV-2 infection. Additional research showed that for ICU patients in the first year of the pandemic, 90–95% of admitted patients were admitted because of COVID-19, and 5–10% were admitted for another reason but tested positive with SARS-CoV-2 in hospital (https://www.stichting-nice.nl/covid-19-faq.jsp). From ISO-week 8 in 2022 onwards, i.e. after Omicron became the dominant strain in the Netherlands, a selection of hospitals started to supply NICE with the indication for admission, related to COVID-19. Since then, depending on the week, between 54% and 78% of SARS-CoV-2-infected patients in these reporting hospitals had COVID-19 as the primary or a secondary reason for admission (https://www.stichting-nice.nl/covid-19-op-de-zkh.jsp). For ICU patients, this percentage ranged between 33% and 81% (https://www.stichting-nice.nl/covid-19-op-de-ic.jsp).

#### Use in open data sets

Three open data sets contain information from the NICE registry only. These concern the daily number of hospital admissions per municipality (Table S1, dataset 8)^22^, the daily number of ICU admissions in the entire country (Table S1, dataset 9)^23^ and the weekly number of hospital and ICU admission by age-group in the entire country (Table S1, dataset 10)^24^. The last two datasets are presented at country-level to anonymize the datasets.

### Number of infectious persons and effective reproduction number (Rt)

#### Data collection

No additional data are collected, these variables are calculated based on both OSIRIS and NICE data.

#### Variable definition

The number of infectious persons is estimated from age-specific seropositivity rates in nationally representative sero-surveys^25^ and age-specific hospital admission data from the NICE database. It was assumed that the age-specific hospitalization probability per seroconverted person was constant, and that an infected person is infectious for on average 8 days, starting 2 days before symptom onset. Because of a low number of hospital admissions between 12 June and 8 October 2020, this estimation of number of infectious persons was informed by the notified case rate in OSIRIS instead of the hospital admission rate. The RIVM ceased to calculate the number of infectious people after 6 July 2021, because, due to vaccination and emergence of a more severe variant, the number of hospital admissions no longer reflected the number of infectious persons.

Since testing only became widely available in June 2020, the R_t_ was calculated based on NICE hospital admissions until 12 June 2020, and based on case notifications thereafter. A published standard method is used^26^, assuming a generation interval of on average 4 days for all pre-Omicron variants and 3.5 days for the Omicron variants^2^. The number of hospital admissions or case notification are nowcasted to account for reporting delays^27^.

#### Use in open datasets

We generate two open datasets with estimated values for (1) the daily number of infectious persons in the Netherlands (Table S1, dataset 11)^28^ and (2) the effective reproduction number (R_t_) (Table S1, dataset 12)^29^.

### SARS-CoV-2 testing data

To satisfy the demand for SARS-CoV-2 testing, all MHSes in the Netherlands have set up large-scale test facilities for free-of-charge PCR and rapid antigen testing for people with COVID-19-like symptoms or those exposed to a SARS-CoV-2 infected person. At its peak capacity in January 2022, this concerned over 350 locations that tested over 150,000 persons per day. The open data neither include the tests done for the purpose of satisfying international travel requirements that were also carried out by these same MHS test facilities, nor do they include tests conducted by commercial testing facilities.

#### Data collection

Appointments for SARS-CoV-2 testing can be made online or through a toll-free number. At the time of booking, clients are asked a number of questions about date of symptom onset, previous contact with other SARS-CoV-2 positive persons, their risk factors for SARS-CoV-2 infection, vaccination status and whether they book a test to confirm a positive result on a self-test. These data thus constitute a source of information both about persons who test positive and those who test negative. The self-reported data entered online by clients or call center staff are stored in a software system called CoronIT (Topicus.com Inc.), under the person’s BSN and sample identifier. As soon as they become available, MHSes and laboratories add the test results and data about the laboratory sample to these client and appointment data, based on the sample identifier.

Daily downloads from this CoronIT test facility system are sent to the RIVM and the RIVM publishes open data about test appointments conducted at least 2 days prior to the publication date. Because of privacy law, the RIVM does not receive the BSN identifier and therefore de-duplicates records based on having the same year of birth, shortened postal code, sex, timestamp for appointment booking (in seconds) and timestamp for appointment attendance (in minutes).

#### Variable definition

Tests performed are defined as test appointments for which a test result is available, whereas positive tests are those with either a positive PCR result or positive rapid antigen test result.

#### Use in open datasets

CoronIT test facility data are reported as the daily total number of tests done and the number of positive test results (Table S1, dataset 13)^30^. To anonymize the dataset it is presented at security region level rather than municipal level.

### National Sewage Surveillance

The RIVM, in collaboration with the 21 Dutch Water Authorities, has published open data on a sewage surveillance system for COVID-19 from 30 March 2020 onwards. This National Sewage Surveillance (“Nationale Rioolwater Surveillance” in Dutch (NRS)) started with 3 sewage sampling locations^4^ and was scaled up to 28 sewage treatment plants (STPs) plus Schiphol airport on 30 March 2020. National coverage was reached on 7 September 2020 with over 300 STPs participating. In the Netherlands, more than 99% of households are connected to the sewer system. Currently, sewage from each of the 313 STPs is analyzed for SARS-CoV-2 four times per week.

#### Data collection

STPs take a minimum of four 24-hr sewage samples per week as follows: a sampling machine takes small sewage samples with a frequency proportional to the flow into the STPs, such that the total sample taken is proportional to the total volume flowing through the STP in the 24 hr period. A half-liter bottle with standardized labeling is then filled from this thoroughly mixed larger sample and stored and transported to the RIVM laboratory in a cold chain complying with international, generic, ISO 5667‐10 standards [ISO] and national, specific, NEN-6600-1 standards^31^ for wastewater sampling by contracted transporters. Ct values are established through qRT-PCR analysis after RNA extraction using the MagNA Pure 96 DNA and Viral NA Large Volume Kit (Roche^TM^). The qRT-PCR is done in duplicate for both the N1 and N2 targets situated on the N gene. Standardized samples with known concentrations are analyzed in parallel, and a moving 7-day average of the resulting calibration data is used to convert the Ct values to RNA particles/mL. The four resulting concentrations (2x N1 and 2x N2) are then averaged to obtain one SARS-CoV-2 concentration per sample. This concentration is multiplied by the 24-hr flow into the STPs to adjust for variation in volume of water (e.g. due to rainfall) to obtain the total absolute viral load. Finally, this viral load is divided by the number of inhabitants in the STPs catchment area and multiplied by 100,000 to arrive at the viral load per 100,000 inhabitants.

Flow data are obtained through the”Z-info” information system of the “Het Waterschapshuis”, a knowledge institute of the Dutch Water Authorities. Through Z-info, data from individual STPs is collated and made available via webservices for approved users such as the NRS. Data access can be requested at “Het Waterschapshuis” by sending an email to servicedesk@hetwaterschapshuis.nl. The number of inhabitants per sewage treatment plant catchment-area is provided by Statistics Netherlands (https://www.cbs.nl/nl-nl/maatwerk/2021/06/inwoners-per-rioolwaterzuiveringsinstallatie-1-1-2021).

The NRS surveillance does not include wastewater processed by STPs owned and run by large industrial estates, however wastewater from businesses that drains onto the public sewage system is included. As of yet, no adjustment for this non-household flow of wastewater is made in the data. The number of inhabitants per STP is static data, i.e. is based on the registered residents in the STP catchment area. Dynamic changes in the number of users of a specific STP, e.g. related to tourism or work mobility are not taken into account.

Viral loads in sewage water cannot yet be used to estimate the number of infected people, because there is still limited published data on shedding in feces^32^ and in urine^33^ which mostly concern symptomatic patients, and a large variation in shedding was found both between infected persons and over the course of their infection^34^. However, re-emergence of SARS-CoV-2 after a period of low or no circulation can be detected using NRS surveillance, as can trends in viral load over time and between locations without being influenced by changes in behavior or policy^35^.

#### Variable definition

Viral concentration in sewage water is defined as the number of virus particles per 100,000 inhabitants of an STP catchment area.

#### Use in open data sets

The number of viral particles per 100,000 persons per STP per day is published as open data (Table S1, dataset 14)^36^. STPs have new samples available for analysis on average 4 times per week, though not all on the same days. Therefore, open datasets are updated on workdays to always present the most recent measurements for all STPs, and on average represent the situation of 3–5 days prior.

### Surveillance of virus variants

Since the start of the pandemic, several laboratories have carried out whole genome sequencing in order to identify virus variants and track their relative contribution to all infections. Since 30 November 2020, collaborating laboratories have consolidated this into a nationally representative variant surveillance system. Initially, sequencing was mostly performed at the RIVM and the Erasmus University Medical Center; later a sequencing network, called SeqNeth, was initiated, which is coordinated by the RIVM.

#### Data collection

Thirty-six laboratories send RNA isolates from randomly selected SARS-CoV-2 positive PCR-tests to the RIVM for whole genome sequencing. These positive samples come from hospitals, large scale MHS testing facilities, and, to a lesser degree, from general practitioners participating in surveillance of influenza-like symptoms (NIVEL). In addition to this, SeqNeth laboratories send genome consensus sequences to the RIVM for typing and inclusion in the surveillance program. The descriptive data (metadata) on these samples, as well as all the consensus sequences, are collected in a single database, Nonacris. These data are subsequently complemented with typing data and are linked to data from OSIRIS using the CoronIT sample identifier, where available.

The RIVM strives to collect approximately 1500 sequences per week, to be able to detect novel variants at 0,5–1,5% prevalence (https://www.ecdc.europa.eu/en/publications-data/guidance-representative-and-targeted-genomic-sars-cov-2-monitoring). The median weekly sample size was 1479 and varied between 75 at the very start of the national surveillance to 2449 samples in the first ISO week of July 2021, which coincided with a sudden peak in infections following abrupt relaxation of non-pharmaceutical interventions against COVID-19. Due to transport and processing time, variant surveillance results are available as open data a median of 15 days after sampling date.

For those sequence reads generated at the RIVM, consensus sequences are derived using the SARS2seq pipeline (https://github.com/RIVM-bioinformatics/SARS2seq/), after which they are manually curated. All sequences are typed using the most recent versions of NextClade^37^ and Pangolin^38^.

#### Variable definition

Sequences are classified as variant of concern (VOC), variant of interest (VOI), variant under monitoring (VUM) and de-escalated variant (DEV) according to WHO (https://www.who.int/en/activities/tracking-SARS-CoV-2-variants/) and ECDC standards (https://www.ecdc.europa.eu/en/covid-19/variants-concern). Due to updates in typing tools, as well as the epidemiological significance of variants, both the assigned variant of a sequence as well as the variant status might change. In these events the data are adjusted retrospectively.

#### Use in open data sets

The total number of samples sequenced, and number of samples per variant per ISO week is published as open data (Table S1, dataset 15)^39^.

### Vaccination coverage

The RIVM has two main datasets about vaccinations, i.e. 1) CIMS and 2) CoronIT.

1. CIMS (COVID-vaccination Information and Monitoring System) is the national registry for COVID vaccination established by the RIVM. Depending on the client group, vaccination is provided by nursing home clinicians, hospital clinicians, general practitioners, and MHSes; the latter are responsible for the bulk of COVID vaccinations given. The RIVM is responsible for quality control and dispatching of vaccines and the monitoring and evaluation of the implementation and effectiveness of the COVID-19 vaccination program. Monitoring of adverse events after COVID-19 vaccination is the responsibility of The Netherlands Pharmacovigilance Centre Lareb (https://www.lareb.nl/en/pages/about-lareb).

#### Data collection

All providers of COVID-19 vaccination submit a core set of individual level vaccination data to CIMS after obtaining permission from their client. These are registered at each vaccination visit and concern the BSN identifier, date of birth, first and last name, residential address, date and place of vaccination, the name and batch number of the vaccine, and type of healthcare professional who provided the vaccination.

An estimated 7% of vaccinated persons does not consent to this data sharing.

2) CoronIT_contains data about all vaccinations given by MHSes. This data is shared with the RIVM as a minimal dataset from the CoronIT platform which includes full information about vaccines given, but only very limited personal information (such as birth year and municipality of residence). This dataset includes vaccinations for which no consent is obtained to be registered in CIMS.

#### Data collection

These data are collected by vaccination center staff employed by MHSes.

#### Variable definition

Partly vaccinated is defined as having received at least one dose of a COVID-19 vaccine within the primary vaccination series. Completely vaccinated is defined as having received the primary series of the COVID-19 vaccine, i.e. one vaccination for the Jcovden vaccine and two for all other vaccines. The exception are persons who were SARS-CoV-2-infected prior to their first vaccination, for whom one vaccination constitutes the completed primary series. Persons who received a vaccine for which one dose constitutes the primary series are thus counted under both definitions. First booster is defined as receiving the first booster vaccination after completing the primary series. Repeat vaccination was offered to persons of 60 years and older, nursing home residents and adults with an immune deficiency or Down syndrome and is defined as receiving a vaccine dose after receiving both primary series and the booster vaccination.

#### Use in open datasets

The CIMS data are used to construct the open dataset on cumulative partial and completed vaccination coverage per neighborhood for persons aged 12 years and above, per calendar week (Table S1, dataset 16)^40^. To preclude identification of individual persons, the vaccination coverage in municipalities with more than one neighborhood with fewer than 60 inhabitants is omitted for those neighborhoods. If just one neighborhood has low population numbers, it is merged with an adjacent neighborhood. Vaccination coverages of < = 5% or > = 95% are not further specified. Denominator data on all inhabitants of 12 years and above are obtained from the CIMS database and based on the Personal Records Database maintained by municipalities.

A second open dataset on vaccination combines the CIMS vaccination data with the CoronIT data (i.e. vaccinations provided by MHSes) for persons who did not give their consent for registration in CIMS. This concerns the open data set on the cumulative partial and completed vaccination coverage for the primary series, for the booster vaccination and for the repeat vaccination for each birth cohort, by municipality and security region (Table S1, dataset 17)^41^. Denominator data on all inhabitants old]er than 12 years of age are obtained from the CBS and based on the Personal Records Database maintained by municipalities. The median time between vaccination being given and data being available in the open data set is 1 day.

### Population-based self-reported symptoms

The “Infectieradar” (Infection radar) is an internet-based participatory surveillance system for COVID-19-like symptoms hosted by the RIVM^42^.

#### Data collection

Voluntary participants submit weekly survey forms about COVID-19-like symptoms and other data, thus allowing estimation of the incidence rate of self-reported COVID-19-like illness. The number of participants per week varied from 6,230 at the start of the surveillance on 1 November 2020 to 13,161 on 18 January 2021 and is on average 10,485 (status per 22 August 2022). Compared to the general Dutch population, females, higher educated people and older age-groups are over-represented in Infectieradar^42^. Reported percentages of people having COVID-19-like symptoms are not adjusted for these divergences from the general population structure. Open data are available from 1 November 2020 onwards, but are missing for the periods 12–19 October 2021, 18–19 June 2022 and 26–27 July 2022 during which time a platform issue led to incomplete data being collected.

#### Variable definition

The incidence rate of COVID-19-like symptoms is defined as the percentage of people out of all participants having fever and/or cough and/or shortness of breath and/or loss of smell and/or loss of taste in the week before they submitted the survey.

#### Use in open data sets

As open data set, both the daily incidence rate of COVID-19-like symptoms is reported and its 7 day-moving average around the reporting date (the 7 days being the 3 days prior, the reporting date itself and the 3 days after the reporting date) (Table S1, dataset 18)^43^.

### Geocoding

Depending on the dataset, counts may be given by neighborhood, municipality or security region, which are presented by name and geocode. As per 1 January 2022 there were 3248 neighborhoods, 345 municipalities and 25 security regions in the Netherlands. Municipalities correspond to the LAU level as specified in the Nomenclature Used for Territorial Statistics (NUTS) maintained by Eurostat. These can used to aggregate to NUTS level 3 and higher (https://ec.europa.eu/eurostat/web/nuts/local-administrative-units). Security regions are part of the local governance structure and are mandated to coordinate the response to fires, disasters and crises, including subnational outbreak control measures. These geocodes can be used to link to the corresponding geographical area polygons using the open dataset on geographical demarcations maintained by the Statistics Netherlands (https://data.overheid.nl/en/dataset/62e15c80-60af-41e2-90de-f5a454230c72).

Every year, municipal reorganization may lead to fusion of one or more municipalities or redrawing of borders between adjacent municipalities, thus causing changes in the population numbers that serve as rate denominators in the Dutch COVID-19 surveillance. In the metadata of each dataset included in Table S1, it is described in detail for which period which municipal demarcations have been applied.

## Data Records

The datasets described in the sections of the methods are available at Overheid.nl (ref. ^13–19,22–24,28–30,36,39–41,43^) and detailed information describing these datasets is available in supplementary table S1. English language metadata can be downloaded from this repository. The datasets are also deposited in two additional repositories, i.e. https://data.rivm.nl/ and https://nationaalgeoregister.nl/. Datasets have permanent identifiers generated using the UUID system and are made available as JSON and in most cases also CSV formats. Extensive metadata descriptions are provided, using the ISO 19115 geodata schema which are downloadable in ZIP, PDF, XML and RDF format and have permalinks. Data are licensed as Creative Commons Public Domain Mark 1.0, i.e. data are not copyrighted (https://creativecommons.org/publicdomain/mark/1.0/deed.en). When changes occur to the structure of an open dataset (most often this concerns a variable definition change to reflect changes in infection control guidelines), a new version number is provided within the dataset, allowing machine detection of these changes. Additionally, within the metadata description, a log of all changes is kept, including the version number.

## Technical Validation

Validation of the data obtained from CoronIT, OSIRIS, NICE and CIMS is largely automated and is part of a framework for Automation of Data Integration, Summarization and Communication (A-DISC). This programming framework has been developed by a multidisciplinary team at the RIVM during the COVID-19 pandemic to allow timely, regular, accurate analysis and reporting of surveillance data of increasing complexity, using the open source software R (https://www.R-project.org/). In summary, it consists of a series of automated functions that (i) prepare the constant and static components of the scripts, such as loading R packages, geographic, population and sewage flow data; (ii) carry out data wrangling, i.e. import, validate, transform and save data for each data source; (iii) summarize and store data in tables, figures and open data sets and (iv) report and disseminate information, e.g. as publicly available epidemiological situation reports (https://www.rivm.nl/en/node/163991) (in Dutch only), webpages (https://www.infectieradar.nl/welcome) and uploads into the ECDC Tessy database (https://www.ecdc.europa.eu/en/publications-data/european-surveillance-system-tessy).

As part of the validation step under ii), the script verifies that data have been recently updated, individual records are complete (e.g. no missingness in birthdate, gender, postal code, date of death, comorbidity among diseased cases younger than 70 years of age) and correct (e.g. dates occur in logical order, unlikely birthdate, notification date or date of death before the pandemic, unlikely high age of cases working in health care, institutionalized cases with reported occupation) and screens for extraordinary events (e.g. mortality among patients younger than 45 years of age, pregnant women, health care workers, educational workers) and unlikely young age (<5 yr) at vaccination. Identified records from these validation steps are stored in an Excel workbook file accessible to the surveillance team at the RIVM for further follow-up and at the same time are sent by secured mail to the external data provider for inspection. To resolve potential errors that the surveillance team cannot correct themselves and/or to confirm that the extraordinary events observed are correct, they contact the responsible external data provider staff by phone or email. External data providers then recheck the patient file, and may contact the reporting physician or patient, after which necessary changes are made in the respective source databases. The corrected databases are imported by the RIVM on the same or the next day using the standard procedures.

Using a separate virus variant surveillance script, the reference laboratory adapts data retrospectively to reflect changes in VOC, VOI, VUM and DEV definitions as and when they occur and publishes these as open data.

Validation of data obtained from the quantitative PCR analysis of wastewater samples by the NRS laboratory is largely automated. The PCR tests are performed in duplicate on both the N1 and N2 targets in the N gene. In order to convert PCR results to concentrations, calibration PCR data for both N1 and N2 is simultaneously obtained with sample PCR data, through analysis of prepared samples with known concentrations. Per run, calibration data is selected based on whether fit results of a log-linear fit on the calibration data meet conditions on the slope and the R^2^ of this fit. By using a 7-day moving average of the selected calibration data, possible single-run deviations on the PCR process are averaged out, while long-term systematic effects are taken into account.

The NRS uses a similar data handling and analysis process as described under the A-DISC paragraph above: after obtaining the concentrations for each individual N1 and N2 result, the resulting RNA/ml values per sample are averaged, followed by normalization steps that correct for flow and population per STP-site. Before reporting, the script verifies the sample is not dated after the date of arrival at the laboratory, or if the combination of sampling date and location already exists in the open data. Furthermore, any values higher than 10^15^ viral particles per 100,000 inhabitants are automatically flagged and manually marked for re-testing.

## Usage Notes

**Fig. 2:**
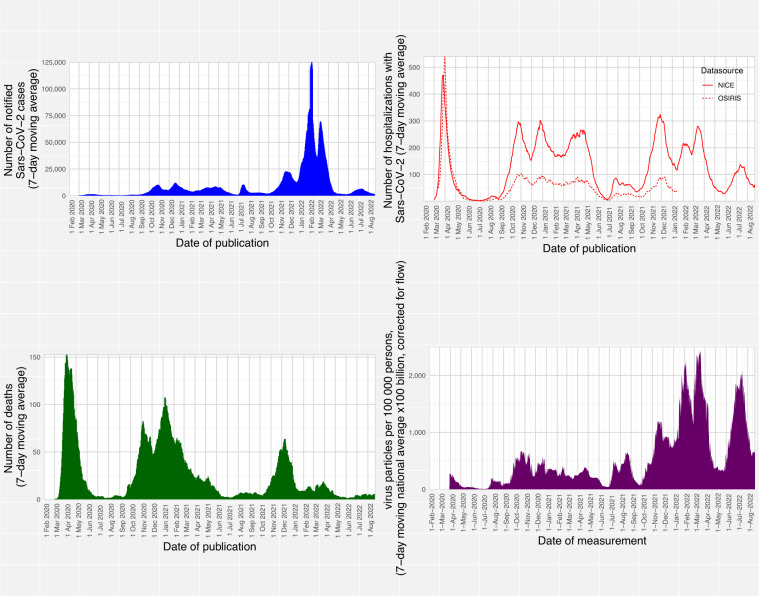
Epidemic curves for four indicators of SARS-CoV-2 infection and disease burden. Number of SARS-C0V-2 cases (upper left panel), hospital admissions (upper right panel), deaths (lower left panel) and sewage water viral load (lower right panel) from the start of the pandemic in the Netherlands (27 February 2020) until 22 August 2022.

**Fig. 3:**
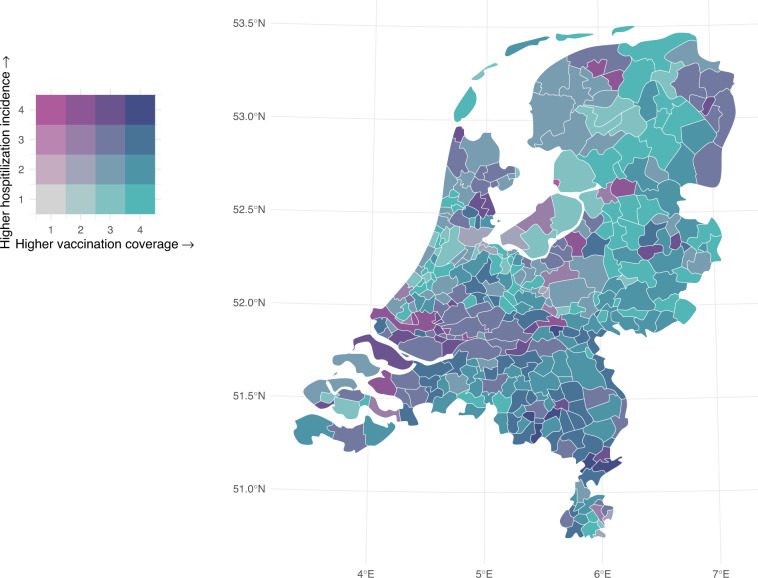
Coverage of minimum 1 vaccine dose among adults per 18 November 2021 (the end of the primary series vaccination campaign) and hospitalisation incidence between 20 January and 2 December 2021 (2 weeks after the start and end of the primary series vaccination campaign). Data are grouped to maximize within-group homogeneity using Fisher-Jenks breaks^45^.

**Fig. 4:**
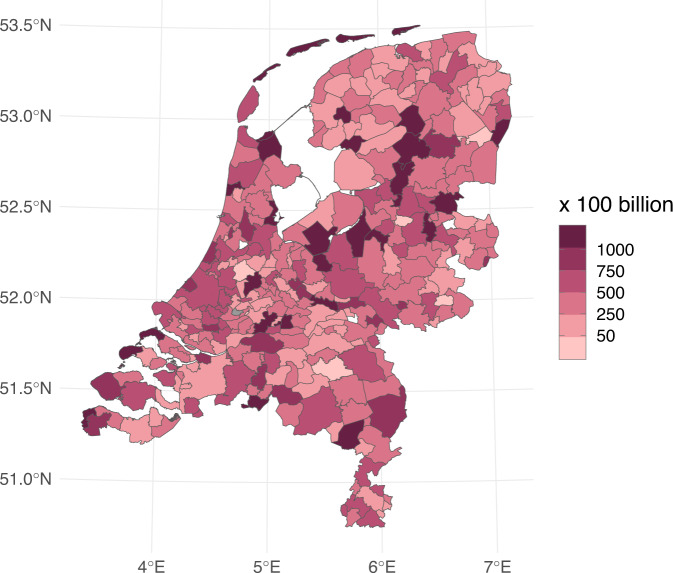
SARS-CoV-2 virus particle concentration in sewage based on the most recent STP measurement within the period 14th -22nd August 2022.

These standardized, validated datasets cover key aspects of the pandemic plus vaccination coverage, are available at detailed geographical level and are provided near real-time, which allows users to gain a detailed understanding of the past and current COVID-19 epidemiology in the Netherlands. These data allow e.g. analysis of trends (Fig. 2) and identification of hotspots of high transmission and disease incidence and low vaccination coverage (Fig. 3) or hotspots of virus excretion (Fig. 4). The resource is used extensively: until the time of writing over 600,000 unique users have downloaded over 11 million open datasets.

The open data are dynamic and are updated on workdays at 15:15 on https://data.rivm.nl/covid-19/. This data descriptor was peer reviewed in 2023 based on data available on the platform at the time. Static versions of datasets from a particular date can be obtained by sending an email to the address specified on the metadata page of the requested dataset. The datasets in CSV format can easily be imported in any analysis or visualization software package, and the JSON files are available for use of data on e.g. webpages. All variable definitions are described in detail in the corresponding metadata pages.

The number of tests performed and the number of cases reported need to be carefully interpreted against the background of the indication setting for testing, the test capacity and a person’s likelihood of testing. For example, from 31 March 2021 until 11 April 2022, a positive self-test result needed to be confirmed by an MHS-test. From 11 April 2022 onwards, this is no longer required. Therefore, the number of people tested at the MHS has decreased substantially. Due to the changed composition in the group of people who get tested at the MHS, the overall positivity rate is also lower. A timeline of test policy and capacity changes in the Netherlands can be found under the figure about positive SARS-CoV-2 tests on the national COVID-19 dashboard (https://coronadashboard.government.nl/landelijk/positief-geteste-mensen) and an analysis of correlates of testing is available from Statistics Netherlands (https://www.cbs.nl/nl-nl/nieuws/2021/34/minder-geteste-mensen-bij-grotere-afstand-tot-ggd-testlocatie). Such variability does not affect hospital admissions, nor virus loads in sewage which can be compared over time and between locations (e.g. STPs, municipalities). However, for the NRS, the use of static population numbers based on residents (instead of actual STP users) in order to calculate the viral load/100,000 population may lead to high apparent viral load densities in popular tourist areas that have a low number of residents (such as the Frisian islands in the North), which was clearly observed during school holiday periods.

The RIVM is currently using these open datasets to further analyse the strong correlation between viral load in sewage and hospitalization incidence (50) and -through linkage with other publicly available datasets- to estimate the environmental and sociodemographic covariates of infection and hospitalization for COVID-19, such as level of urbanization, proximity to livestock farming^44^, air pollution levels, age structure and family size. Future work could e.g. focus on in-depth country-comparative analysis on the effect of non-pharmaceutical interventions against COVID-19 (such as summarized in the Oxford Policy indicators (https://www.bsg.ox.ac.uk/research/research-projects/covid-19-government-response-tracker) in different contexts, such as different cultural values or levels of socio-economic disparity, COVID-19 and climate factors.

There are some limitations in the use of these open datasets. Since all concern aggregated data, apart from the case-based register that provides only a few case characteristics, analysis of individual-level risk factors for infection, disease or death is largely impossible. Likewise, estimation of individual-level correlates of vaccination uptake is not feasible. Lastly, detailed transmission modelling is precluded with these datasets.

In line with General Data Protection Regulation^1^, linkage of the open datasets with external data on e.g. health care use, participation in the workforce or the education system and presence of underlying disease is not possible, thereby prohibiting their use for studies by external parties on underlying disease-dependent differences in vaccine-efficacy, long-term health consequences of a SARS-CoV-2 infection or hospitalization, and societal impact of COVID-19. The RIVM, under its mandate of surveillance for public health protection, does have the legal authorization to linked datasets that allow some of the above analyses.

Lastly, because case notification data are known to be incomplete with regards to registration of deaths due to COVID-19, alternative methods of calculating the mortality impact of SARS-CoV-2 are advised, i.e. excess mortality estimation and cause-specific mortality analysis (https://www.cbs.nl/nl-nl/longread/rapportages/2022/sterfte-en-oversterfte-in-2020-en-2021).

As COVID-19 gradually becomes endemic, the national surveillance systems will be adapted as well to allow sustainable monitoring of SARS-CoV-2 to detect future changes in its virology or epidemiology that may once again lead to an increased disease burden. Where these changes in systems have impact on the availability or interpretation of open datasets, they will be communicated as part of the metadata pages.

## Supplementary information

Overview of COVID-19 open data sets

## Data Availability

While custom code was utilized to generate this dataset from source data, this code specifically incorporates analysis of personal data collected by various governmental programs which cannot be shared. No custom code is necessary to utilize the published collated datasets.
