# Supplementary material for: The daily updated Dutch national database on COVID-19 epidemiology, vaccination and sewage surveillance
Source: Sci Data. 2023 Jul 20;10:469. doi: 10.1038/s41597-023-02232-w (PMC10359398; doi:10.1038/s41597-023-02232-w)
Supplement: Supplementary file 1 — Overview of COVID-19 open data sets [file 41597_2023_2232_MOESM1_ESM.pdf]

Supplementary Table 1. Datasets, variables, data sources, version number of the dataset as described in this Data Descriptor and permanent links for the 18 open datasets on COVID-19 epidemiology

Ref	Open data set	Contents	Data sources	Version number	Notes	Permanent link	Metadata unique identifier
13	1. Case-based register (29/05/2020-present)	Version, Date_file, Date_statistics, Date_statistics_type, Agegroup, Sex, Province, Deceased, Week of Death, Municipal_health_service	OSIRIS	5		https://data.overheid.nl/dataset/34ff61d8-17a6-49a6-94db-90826562f8b5	2c4357c8-76e4-4662-9574-1deb8a73f724
14	2. Cumulative number of notified, (hospitalized) and deceased cases per municipality per day (30/04/2020-present)	Version, Date_of_report, Municipality_code, Municipality_name, Province, Total_reported, Hospital_admission, Deceased	OSIRIS	6	Hospital admissions no longer updated from 18 January 2022 onwards, instead refer to open data set 8	https://data.overheid.nl/dataset/dce48c27-2b1c-40c4-9ec0-bb9f9ddc62ac	1c0fcd57-1102-4620-9cfa-441e93ea5604
15	3. Number of new cases, (hospital admissions) and deaths per municipality per day (20/08/2020-present)	Version, Date_of_report, Date_of_publication, Municipality_code, Municipality_name, Province, Security_region_code, Security_region_name, Municipal_heath_service, ROAZ_region, Total_reported, Hospital_admission, Deceased	OSIRIS	5	Hospital admissions no longer updated from 18 January 2022 onwards, instead refer to open data set 8	https://data.overheid.nl/dataset/357b5f72-b13d-402c-932b-4ff476703a1a	5f6bc429-1596-490e-8618-1ed8fd768427
16	4. Number of new cases and deaths in disability care institutions and number of locations with new and ongoing outbreaks, per security region per day (09/12/2020-present)	Version, Date_of_report, Date_of_statistic_reported, Security_region_code, Security_region_name, Total_cases_reported, Total_deceased_reported, Total_new_infected_locations_report	OSIRIS	4		https://data.overheid.nl/dataset/a0ccf016-ec08-4691-ad1a-b39f757e1c8c	5f6bc429-1596-490e-8618-1ed8fd768427

		ed, Total_infected_locations_reported					
17	5. Number of new cases and deaths in nursing homes and number of locations with new and ongoing outbreaks, per security region per day (06/11/2020-present)	Version, Date_of_report, Date_of_statistic_reported, Security_region_code, Security_region_name, Total_cases_reported, Total_deceased_reported, Total_new_infected_locations_reported, Total_infected_locations_reported	OSIRIS	5		https://data.overheid.nl/dataset/aea2201e-1e65-489d-9cdb-d7fa5305ccbd	0179dd26-7bf6-4021-857f-8623aaf8e73a
18	6. Number of new cases and deaths among elderly (70 years and older) living at home, per security region per day (09/12/2020-present)	Version, Date_of_report, Date_of_statistic_reported, Security_region_code, Security_region_name, Total_cases_reported, Total_deceased_reported	OSIRIS	5		https://data.overheid.nl/dataset/dc728b21-da08-4e96-a6e2-1e492df13567	40508e17-7296-4f39-ad25-8ddd0c904087
19	7. Possible settings of transmission of newly notified cases, per security region per day (09/06/2021-present)	Version, Date_of_report, Date_of_publication, Security_region_name, Security_region_code, Source_and_contact_tracing_phase, Total_reported, Reports_with_settings, Setting_reported, Number_settings_reported,	OSIRIS	5		https://data.overheid.nl/dataset/bc607546-ba52-470e-90da-a7366bad3966	40508e17-7296-4f39-ad25-8ddd0c904087
22	8. Number of hospital admissions per municipality per day (17/12/2020-present)	Version, Date_of_report, Date_of_statistics, Municipality_code, Municipality_name, security_region_code, Security_region_name,	NICE	5		https://data.overheid.nl/dataset/76838483-4d36-47d4-8aad-491d749c902b	40508e17-7296-4f39-ad25-8ddd0c904087

		Hospital_admission_notification, Hospital_admission					
23	9. Number of ICU admissions per day (18/03/2021-present)	Version, Date_of_report, Date_of_statistics, IC_admission_notification, IC_admission	NICE	2		https://data.overheid.nl/dataset/9de57060-e9f3-4011-8b38-5ca6b4e68cad	40508e17-7296-4f39-ad25-8ddd0c904087
24	10. Number of hospital and ICU admission, per age-group per week (28/04/2021-present)	Version, Date_of_report, Date_of_statistics_week_start, Age_group, Hospital_admission_notification, Hospital_admission, IC_admission_notification, IC_admission	NICE	2		https://data.overheid.nl/dataset/8c5d3341-2aaf-4186-af6d-612f06ad0ae7	40508e17-7296-4f39-ad25-8ddd0c904087
28	11. Estimated number of infectious persons, per day (01/09/2020-06/07/2021)	Version, Date, prev_low, prev_avg, prev_up, population	OSIRIS, NICE	1	Discontinued per 6 July 2021	https://data.overheid.nl/dataset/c6b43515-c3f7-4eb6-9bd5-daccdc6eec31	40508e17-7296-4f39-ad25-8ddd0c904087
29	12. Estimated effective reproduction number (Rt), per day (23/06/2020-present)	Version, Date, Rt_low, Rt_avg, Rt_up, population	OSIRIS, NICE	3		https://data.overheid.nl/dataset/ef74e3f5-2c56-4c4f-93cd-e06f3b4db168	40508e17-7296-4f39-ad25-8ddd0c904087
30	13. Number of tests performed and positive, per security region per day (05/02/2021-present)	Version, Date_of_report, Date_of_statistics, Security_region_code, Security_region_name, Tested_with_result, Tested_positive	CoronIT test data	3		https://data.overheid.nl/dataset/fe88e46b-8596-472d-af11-46b9a8748ba5	40508e17-7296-4f39-ad25-8ddd0c904087
36	14. Number of viral particles per 100,000 persons, per sewage	Version, Date_of_report, Date_measurement, RWZI_AWZI_code,	NRS	2		https://data.overheid.nl/dataset/71c27a4a-638d-4e8a-a48d-2e5cc9df38da	40508e17-7296-4f39-ad25-8ddd0c904087

	treatment plant per day (22/06/2021-present)	RWZI_AWZI_name, RNA_flow_per_100000					
39	15. Number of samples sequenced, and number of samples per variant found per ISO week (04/03/2021-present)	Version, Date_of_report, Date_of_statistics_week_start, Variant_code, Variant_name, ECDC_category, WHO_category, May_include_samples_listed_before, Sample_size, Variant_cases,	Nonacris, OSIRIS	4		https://data.overheid.nl/dataset/e8754ba4-8969-4189-ba1f-eee6e52e208e	40508e17-7296-4f39-ad25-8ddd0c904087
40	16. Cumulative partial and completed vaccination coverage, coverage for 1st booster and repeat vaccinations per neighborhood for persons aged 12 years and above, per calendar week. (08/02/2022-present)	Version, Date_of_report, Date_of_statistics, Region_level, Region_code, Region_name, Birth_year, Populatie, ID_koppelcode, Populatie_merged, Coverage_primary_partly, Coverage_primary_completed, Coverage_first_booster, Coverage_repeat_vaccination	CIMS	2	Omitted for neighborhoods with fewer than 60 inhabitants, unless this concerns just one neighbourhood, in which case it is merged with an adjacent neighborhood. Vaccination coverages of <=5% or >=95% are not further specified.	https://data.overheid.nl/dataset/5ff42754-42ff-47c0-ad4b-d857fb736bfa	45152ec7-8d14-46f0-a3e6-4aa9193cc242
41	17. Cumulative partial and completed vaccination coverage, coverage for 1st booster and repeat vaccinations for each birth cohort, by municipality and security region, per calendar week (08/09/2021-present)	Version, Date_of_report, Date_of_statistics, Region_level, Populatie, Region_code, Region_name, Birth_year, Coverage_primary_partly, Coverage_primary_completed, Coverage_first_booster, Coverage_repeat_vaccination, Age_group	CIMS, CoronIT vaccination data	6		https://data.overheid.nl/dataset/a1ef9334-5ab5-405c-856a-9a97b5d49135	45152ec7-8d14-46f0-a3e6-4aa9193cc242

43	18. Daily incidence rate of COVID-19-like symptoms and its 7 day-moving average around the reporting date (14/09/2021-present)	Version, Date_of_report, Date_of_statistics, Perc_civid_symptoms, MA_perc_COVID_symptoms	Infection radar	1		https://data.overheid.nl/dataset/0d220bef-a12f-4a0a-aea1-9a8b5acd9f2c	7bd9dfbf-6b30-47a4-a279-fee4a0964400
